# An ensemble learning method with GAN-based sampling and consistency check for anomaly detection of imbalanced data streams with concept drift

**DOI:** 10.1371/journal.pone.0292140

**Published:** 2024-01-26

**Authors:** Yansong Liu, Shuang Wang, He Sui, Li Zhu

**Affiliations:** 1 School of Software Engineering, Xi’an Jiao Tong University, Xi’an, Shaanxi, China; 2 School of Intelligent Engineering, Shandong Management University, Jinan, Shandong, China; 3 Information Security Evaluation Center of Civil Aviation, Civil Aviation University of China, Tianjin, China; 4 College of Aeronautical Engineering, Civil Aviation University of China, Tianjin, China; University of Sindh, PAKISTAN

## Abstract

A challenge to many real-world data streams is imbalance with concept drift, which is one of the most critical tasks in anomaly detection. Learning nonstationary data streams for anomaly detection has been well studied in recent years. However, most of the researches assume that the class of data streams is relatively balanced. Only a few approaches tackle the joint issue of imbalance and concept drift. To overcome this joint issue, we propose an ensemble learning method with generative adversarial network-based sampling and consistency check (EGSCC) in this paper. First, we design a comprehensive anomaly detection framework that includes an oversampling module by generative adversarial network, an ensemble classifier, and a consistency check module. Next, we introduce double encoders into GAN to better capture the distribution characteristics of imbalanced data for oversampling. Then, we apply the stacking ensemble learning to deal with concept drift. Four base classifiers of SVM, KNN, DT and RF are used in the first layer, and LR is used as meta classifier in second layer. Last but not least, we take consistency check of the incremental instance and check set to determine whether it is anormal by statistical learning, instead of threshold-based method. And the validation set is dynamic updated according to the consistency check result. Finally, three artificial data sets obtained from Massive Online Analysis platform and two real data sets are used to verify the performance of the proposed method from four aspects: detection performance, parameter sensitivity, algorithm cost and anti-noise ability. Experimental results show that the proposed method has significant advantages in anomaly detection of imbalanced data streams with concept drift.

## Introduction

The joint of class imbalance and concept drift is a crucial issue for anomaly detection in real-world application, where the underlying data distribution changes over time [1]. On one hand, the data categories are not equally represented, which can cause learning bias towards the majority class and result in poor generalization [2]. On the other hand, concept drift is also a challenging problem in the field of data streams learning [3], which deeply influences the stability of data streams classification. Unfortunately, most of data streams are characterized as high speed, nonstationary data distribution, and infinite length, facing the joint of imbalance and concept drift. This may further hinder the anomaly detection, then the high demand for this type of solution is evident [4].

Class imbalance is mainly reflected in the following three aspects [5]: 1) great difference in data volume; 2) imbalanced distribution of samples; 3) overlap of samples in feature space. As a result, little anomaly data is always buried in a sea of normal data [6]. Therefore, how to overcome class imbalance and obtain high detection accuracy is an urgent problem to be solved for anomaly detection [7]. Generally, solutions are tried mainly from three strategies: data processing, feature extraction and algorithm optimization. For data processing, sampling has been carried out to improve the balance of dataset and change the samples’ distribution [8]; for feature extraction, an appropriate feature model is used to map the data to the feature space, and better feature representation is obtained [9]; for algorithm optimization, optimized algorithm is applied to improve the recognition rate of minority class samples [10]. Obviously, data-level approaches are easier with no need for understanding of loss function, such as oversampling [11]. Oversampling needs sufficient information to generate high-quality data. However, it is harder to capture the distribution of minority samples, so as to likely to generate low-quality samples deviating from its distribution. Therefore, how to produce more diverse and high-quality samples is still critical.

Due to concept drift, the dynamic of target concepts deteriorates the performance of classifiers learned from past instances. Thus, concept drift requires classifiers to be adjusted to adapt to the new condition. Essentially, concept drift is the change of the joint probability distribution of data [12]. Based on Bayesian theory, the reason of concept drift may be the change of unconditional probability, conditional probability, or both of them [13]. From this perspective, concept drift is divided into virtual drift and real drift [14]. Furthermore, according to the evolution process, concept drift can also be classified as abrupt drift, incremental drift, gradual drift, and recursive drift [15]. The adaptation of concept drift is its vital way to overcome this issue, including methods of model adjustment [16], model reconstruction [17], and ensemble learning [18]. Model adjustment approaches adjusted model parameters based on the transformation of data stream characteristics to adapt to the new data distribution, so as to overcome concept drift. In contrast, when the concept drift is detected, the original model is completely abandoned by model reconstruction approaches, and the detection model is retrained based on the data distribution at this time. Since each detection model has different sensitivity to different types of concept drift, ensemble methods were proposed. It is focus on ensuring the overall detection effect, rather than whether the concept drift of a single model, based on the detection results of multiple models. However, how to avoid overfitting and adjust to more types of concept drift is still challenging for ensemble model.

The problem becomes more complicated if concept drift occurs together with class imbalance, because they will tend to affect each other significantly [19]. For example, traditional drift detection algorithms based on classification error that are insensitive to class imbalance will become inefficient because they cannot detect concept drifts in the minority class. In addition, methods that apply sampling to address class imbalance will be influenced because the imbalance status varies in the case of concept drift with a variable imbalance ratio [20]. To date, only a few methods have been proposed to address the joint of class imbalance and concept drift [21]. Based on the number of processed instances at a time, the existing approaches for tackling the joint problem can be categorized as online algorithms [22] and chunk-based algorithms [23]. However, chunk learners require a large amount of data to build the model. To overcome the issue of class imbalance, chunk-based models need to collect minority data in past chunks and propagate them into the latest chunk. As a part of data items seen in previous batches should be preserved and accessed, this framework is not strictly incremental. Meanwhile, chunk learners cannot handle the real drift, especially when minority samples become the majority class. So incremental learning maybe a better choice. Incremental learning method learns new knowledge while retaining the vast majority of previously learned knowledge. It first uses part of the existing data to build an initial model, and then uses the subsequent data to adjust and update the model to adapt to the changes in the data distribution. Single classifier needs to constantly adjust the internal structure or parameters to adapt to the change of data flow in incremental learning, so it often performs very unstable. The introduction of ensemble learning effectively improves the ability of incremental learning [24, 25].

This paper focus on binary classification because this setup is the most frequently studied in the literature and most commonly meets real-world anomaly detection problems. Our research aims to propose effective data stream anomaly detection algorithms in a situation where a disproportion in the quantity of the arriving objects from different classes is present, together with multifarious concept drift. In this paper, we proposed an ensemble learning method with generative adversarial network-based sampling and consistency check (EGSCC). And the main contributions are as follows:

- We tackled the joint challenges of mixed drift and variable imbalance ratios, and formulated the anomaly detection framework for non-stationary imbalanced data streams, which employs GAN (Generative Adversarial Network)-based oversampling preprocessing, stacking ensemble learning and consistency check technique.
- We introduce encoder into the GAN model and optimize its subnet with SE (Squeeze and excite) block module to better capture the distribution characteristics for overcoming imbalance. Class label is treated as auxiliary information to improve multi-flow pattern learning ability and avoid mode collapse. Wasserstein distance has also been used to measure the distribution distance with gradient penalty and strengthen Lipschitz restriction, which accelerates model convergence and alleviated gradient disappearance.

- We undertake consistency check of incremental instances with the check set. Incremental instance anomaly is detected by statistical learning method, that is the percentile of incremental instance in the validation set is used instead of threshold. Besides, the validation set is dynamic updated according to this percentile result.

The outline of this article is as follows. in Section 2, we review the related research on the composition issue of concept drift and class imbalance. Then, the proposed approach is described in detail in Section 3, and Section 4 presents the experiments and discussions. Section 5 concludes the paper and offers insight into future directions in the field of imbalanced data stream preprocessing.

## Related works

### Anomaly detection for imbalanced data

Anomaly detection for imbalanced data has been extensively studied in recent decade, with several outstanding survey papers published to provide a bird’s eye view [26, 27]. From these surveys, most of state-of-the-art approaches have addressed this problem from aspect of resampling preprocessing, including oversampling and under-sampling. However, under-sampling is faced with the dilemma of deleting sample selection. How to reserve more valuable data is still challenging, because of the parameter sensitivity when evaluating the samples’ value. In addition, the number of deleted samples is always set based on experience, leading to unstable performance. Therefore, it comes as no surprise that oversampling remains by far the more popular method.

The most classic oversampling method is random synthetic minority oversampling technique (SMOTE), which generates samples with linear inter-potation. Despite its effectiveness, recent studies reported that its degeneration is usually associated with noisy. Therefore, several variants based on SMOTE have been developed. Larger weights are given to the hard-to-learn class, and more samples are generated. Noise-immunity majority weighted minority oversampling technique (NI-MWMOTE) [28] starts by adaptively removing noise. Subsequently, it clusters the samples and adaptively determines the number of generated samples using misclassification error. Moreover, a kernel subspace self-organizing map is introduced in Minority oversampling in kernel adaptive subspaces (MOKAS) [29], so as to improve the quality of generated samples.

Despite the above merits, most of these algorithms focus rather on the data characteristic, while they cannot accurately learn the distribution, so that the generated samples lack diversity. In recent years, GAN model has been developed for sampling, given its better ability to fit the data distribution. Engelmann et al. [30] employed conditional GAN combined with auxiliary information to model continuous and class variables to sample data of the specified class. The sampling results were used as the training set for the classifiers. And it is effective to maximize the classification performance on strongly nonlinear datasets. Zheng et al. [31] further introduced the penalty coefficients into the GAN model, which was more comprehensive for data processing and showed greater advantages in terms of data generation quality and model stability. To effectively apply this approach for high-dimensional features, Liu et al. [32] proposed a GAN and feature selection-based method (GAN-FS). The stability and diversity of the generation were achieved using a penalty coefficient to limit the gradient range and further model the complex high-dimensional generated data. Dlamini and Fahim [33] put forward GAN with KL-divergence. This method not only guide learning toward the minority class, but also overcome gradient vanish.

### Anomaly detection for stream data with concept drift

The purpose of either qualitative detection or quantitative analysis for concept drift is to provide a basis for decision making and optimization of concept drift adaptation, so the adaptation of concept drift is its essential problem, including the methods of model adjustment, model reconstruction, and ensemble learning.

When concept drift occurs, finding the drift point and correct it may be the most direct idea to adapt to concept drift. Subtree node adjustment can also be used to achieve model local update. Dynamic extreme learning machine (DELM) dynamically adjusted the number of nodes in the decision subtree based on error rate detection [34]. Besides, the linear classifier can also be used for local adjustment of the model [35]. The principle of local adjustment of the model is the same as weight adjustment of the linear classifier, which is to further amplify the significant influences and focus more on the data features that have a significant impact on the results, while further neglecting to reduce the non-significant influences and weaken their features.

Model reconstruction is to reconstruct the detection model based on all historical data after identifying the concept drift, which can always maintain good detection accuracy. However, frequent model reconstruction will reduce the utilization of the model, putting great pressure on computation and memory of the computing environment. The classical model reconstruction method is adaptive sliding window (ADWIN) algorithm [36]. It was based on the idea of "replacement", in which two models were built in advance to detect and classify incremental instances.

Ensemble learning is a common method to deal with concept drift. The accuracy updated ensemble (AUE) algorithm was proposed by Brzezinski and Stefanowski [37]. On one hand, the variability of each base classifier in the ensemble model is enhanced by a weighting strategy of base classifiers to accommodate abrupt drift. On the other hand, ensemble selection based on sub-classification accuracy is performed to improve the ensemble model to adapt to the gradual drift and obtain better multi-category drift adaptation.

### Anomaly detection for imbalanced data with concept drift

In recent years, the coupling problem of data imbalance and concept drift has attracted more and more attention. Some researchers have proposed algorithms considering these two issues comprehensively.

Zhang et al. [38] combined the reinforcement learning with ensemble model to significantly improved the anomaly detection performance for drifting imbalanced data streams. Its online active learning strategy with interactive interrogation mechanism is used to transform the original unsupervised learning into supervised learning, making it more prominent in anomaly detection with data label unknown or missing. Liu et al. [19] proposed a comprehensive active learning method for multiclass imbalanced streaming data with concept drift. They developed a comprehensive online active learning framework that includes an ensemble classifier, a drift detector, a label sliding window, sample sliding windows and an initialization training sample sequence. And a variable threshold uncertainty strategy based on an asymmetric margin threshold matrix is designed to comprehensively address the problem that a given class can simultaneously be a majority to a given subset of classes while also being a minority to others. A novel sample weight formula that comprehensively considers the class imbalance ratio of the sample’s category and the prediction difficulty is also used. Klikowski and Woźniak [39] presented a novel deterministic sampling classifier with weighted bagging (DSCB) algorithm employs data preprocessing methods and weighted bagging technique to classify non-stationary imbalanced data stream. It builds models based on an incoming data chunk, but it also takes previously arrived instances into account. The proposed approach outperformed state-of-the-art methods on real and artificially generated data streams with various imbalance ratios, label noise levels, and concept drift types. Li et al. [40] proposed a chunk-based incremental ensemble algorithm called dynamic updated ensemble (DUE) for learning imbalanced data streams with concept drift. It learns one chunk at a time without requiring access to previous data, and emphasizes misclassified examples in the model update procedure. As a result, it can timely react to multiple kinds of concept drifts and adapt to the new condition when switching majority class to minority class, without consuming plenty of memory usage. Zyblewski et al. [41] proposed a novel framework employing stratified bagging for training base classifiers to integrate data preprocessing and dynamic ensemble selection methods. Four preprocessing techniques and two dynamic selection methods, used on both bagging classifiers and base estimator levels, were considered. Experimental results carried out on 135 artificially generated data streams proved it outperformed online and chunk-based state-of-art methods.

## The proposed approach

### Approach framework

**Fig 1:**
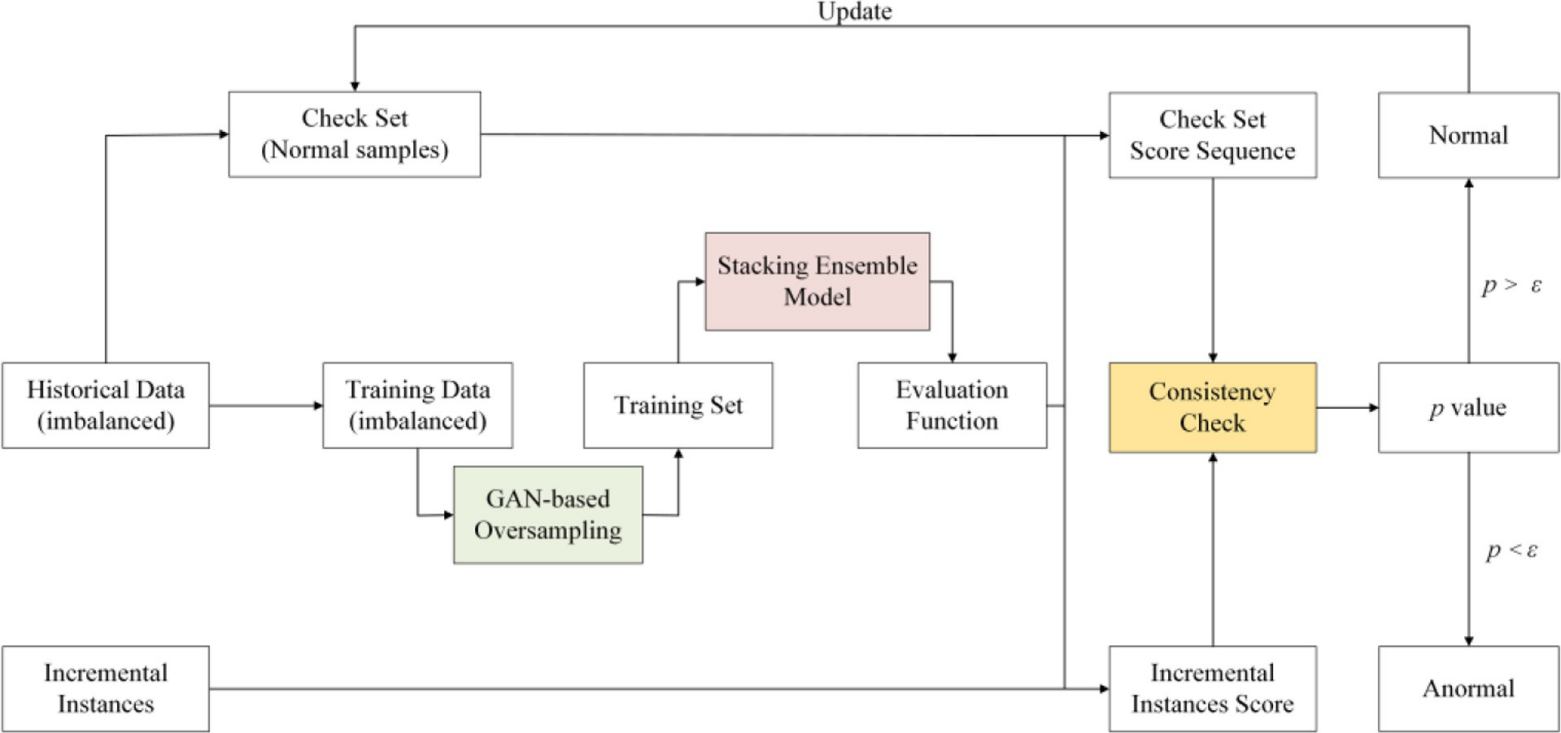
Framework of the proposed EGSCC approach.

The proposed EGSCC approach consists of three essential modules to construct anomaly detection of imbalanced data streams with concept drift (Fig 1).

In the first phase, we extract some of the raw historical data as check set, which are all normal samples. And the other of the imbalanced data are oversampled by an optimized GAN model to achieve rebalancing. In the second phase, we use a stacking ensemble model to obtain an evaluation function to score validation set and the incremental instances. The score value is in [0,1]. Then in the third phase, we take consistency check to implement anomaly detection, with credibility theory of statistical learning, instead of the preset threshold. In addition, we also update the check set according the statistical learning result of consistency check.

### GAN-based oversampling

The data input to the GAN model is the original imbalanced data including majority and minority class data. And the output is some minority class data. Its role is to supplement the minority class samples to overcome the impact of data imbalance on subsequent predictor training.

**Fig 2:**
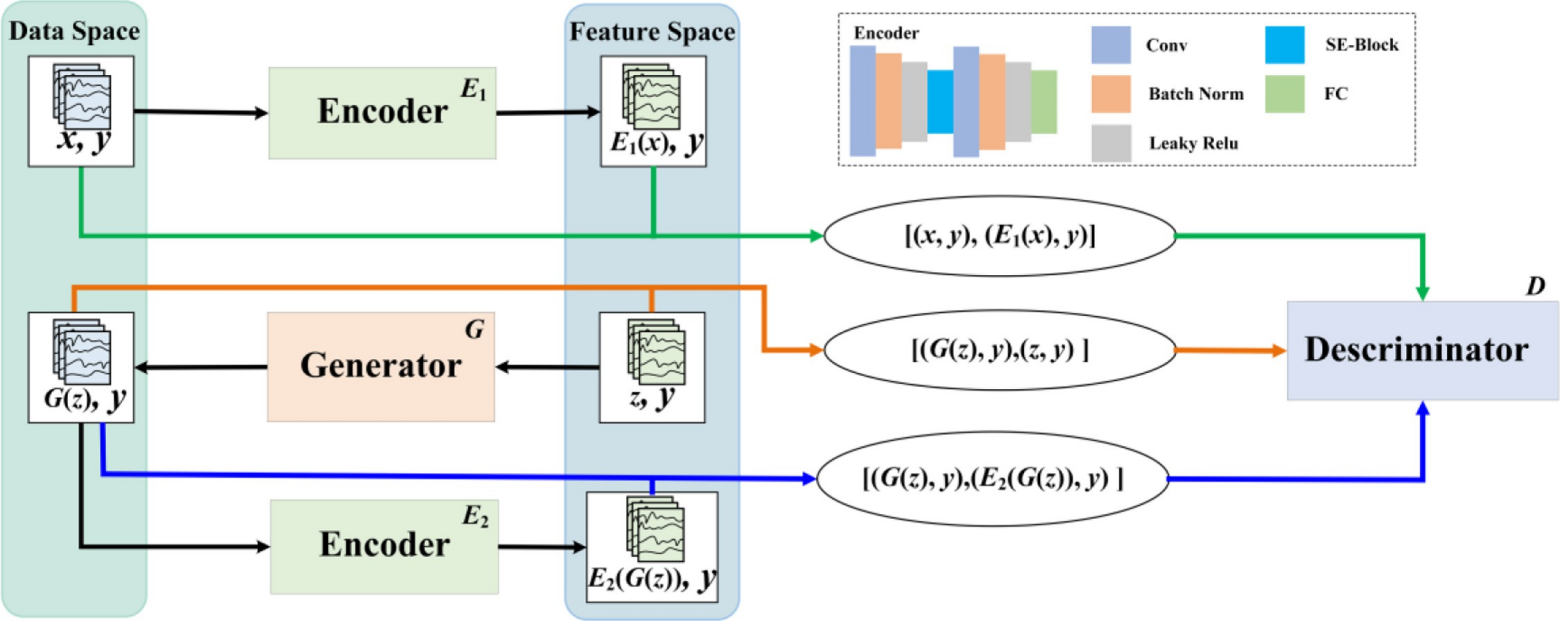
Structure of the GAN model for oversampling.

The structure of the GAN model designed in this paper for oversampling is shown in Fig 2. Double encoders are added to realize the mapping learning from feature space to data space, together with the reconstruction of hidden space features of generated data. Encoders are designed with eight layers, including convolution, Bach Norm, Leaky ReLu, squeeze and excite (SE), and full connection (FC). The convolution layer ensures that the connection can extract the features more effectively, and map low- to high-dimensional space for oversampling operation. Hence, oversampling can improve the resolution of the model to the feature, and achieve higher accuracy through learning. The Bach Norm accelerates the convergence rate of the model and effectively avoids gradient disappearance. The scaling factor within the Bach Norm can effectively identify neurons that contribute little to the network, and some neurons can be automatically weakened or eliminated after the activation function. The Leaky ReLu is used as the activation function, which will count the part of the input that is less than 0. Then the sawtooth problem in the gradient direction is avoided in the backpropagation process. The squeeze and excite (SE) block adopt feature recalibration strategy by feature compression, excitation and reweighting. So as to the weight of effective features is increased, and the weight of invalid or small effect is reduced. The full connection (FC) layer plays the role of “classifier”. By integrating feature representation into a value, it reduces the influence of feature location on the classification results, and improves the robustness of the entire network. As a result, GAN with encoders can learn the characteristics of sample data in the feature space, simplify the data representation, and then obtain effective patterns, further improving the generation ability.

In fact, GAN is a distribution registration transformation operation. Its generator transforms a Gaussian distribution (or other random prior distribution) into a target data distribution. For this purpose, the generator needs to be able to fully mine the characteristics of the data. Encoders are added to further improving the data generation ability. We adopt the feature re-calibration strategy in its subnet structure o to further improve ability of feature learning. It is designed with an eight-layer structure. The squeeze and excite block module embed into the encoder. The weight of effective features is increased, and the weight of invalid or small effect is reduced. By integrating feature representation into a value, it reduces the influence of feature location on the results and improves the robustness of the entire network. With encoders, the generators can better learn high-value features and reappear the distribution of the original data.

The sample (*x*, *y*) is encoded by the encoder *E*_1_ in the data space, and the feature (*E*_1_(*x*), *y*) corresponding to the sample (*x*, *y*) in the feature space is obtained, where *y* is the class label of the data *x*. At the same time, the random noise (*z*, *y*) is decoded by the generator *G* in the feature space, and the generated sample (*G*(*z*), *y*) is obtained. The noise dataset (z, y) generated randomly, which is a Gaussian random variable. In fact, GAN is a distribution registration transformation operation. Its generator transforms a Gaussian distribution (or other random prior distribution) into a target data distribution.

Then the generated sample (*G*(*z*), *y*) is encoded by the encoder *E*_2_ again to obtain the reconstructed feature *E*_2_(*G*(*z*)) of the generated data in the feature space. Thus, three data pairs consisting of data space and corresponding feature space features are generated. Discriminator *D* obtains the discriminant loss by discriminating these three data pairs, and gradually solves the model gradient recursively based on the discriminant loss, so as to realize the update of model weight and reverse optimization of encoder *E*_1_, *E*_2_ and generator *G*.

The number of input neurons for the generator and discriminator is initially set and fixed based on the data feature dimensions to be processed. And the dimensionality of *G*(*z*) and *E*_2_(*G*(*z*)) the same in the feature space. Besides, the dimensionality of *x* and *G*(*z*) are also the same in date space.

The training goal of this model is to achieve a maximum and minimum binary game balance:

$$\underset{G,E_{1},E_{2}}{\min}\underset{D}{\max}V(D,E_{1},E_{2},G)$$ (1)

In the training process, gradient disappearance and model collapse are two main problems for GAN-based model. If the model’s confrontational training reaches the optimal state, the generated and real data distribution are low-dimensional manifolds, and their spatial overlap measure is 0 by *JS* divergence. It means that *G* can only learn a few manifolds, and the distribution *P*_*g*_ of the generated data cannot completely match the distribution *P*_*r*_ of the real data. Under this situation, no matter how far apart the two distributions are from, the divergence of *JS* remains unchanged, thus losing the ability to measure the distribution distance. The update gradient of *G* is 0, so parameters cannot be updated, which is a typical gradient disappearance problem.

To overcome the above problem, Wasserstein distance with better smoothness was instead of *JS* divergence in this paper. Comparatively, the superiority of Wasserstein distance is that even if there is no overlap between the two distributions, it can still reflect their distance. It is calculated as follows:

$$W(P_{r},P_{g})=\underset{\gamma \in \Pi (P_{r},P_{g})}{\inf}{\text{E}}_{(x_{1},x_{2}):\gamma }[||x_{1}-x_{2}||]$$ (2)

Where, inf stands for the lower bound. ∏ (*P*_*r*_, *P*_*g*_) represents the set of the combined distributions of *P*_*r*_ and *P*_*g*_. If two samples *x*_1_ and *x*_2_ are from(*x*,*y*)∼γ, then it calculates the distance between them. Further, the expected value **E**_(*x*_1_,*x*_2_)∼γ_[||*x*_1_-*x*_2_||] of the sample pair’s distance under the joint distribution is obtained, and the Wasserstein distance is obtained as the lower bound of the expected value in all possible joint distributions. Since the Wasserstein distance cannot be solved directly, it is converted to the following form:

$$W(P_{r},P_{g})=\frac{1}{K}\underset{{||f||}_{L}\leq K}{\sup}{\text{E}}_{x∼P_{r}}[f(x)]-{\text{E}}_{x∼P_{g}}[f(x)]$$ (3)

Where, || *f* ||_*L*_ is the additional boundary function, which updates the parameter clipping to a limited range, thus overcoming gradient disappearance. It also adds a Lipschitz constant to the function *f* with the use of repruning, that is, for a continuous function, *K*≥0, the maximum absolute value of the derivative in a certain interval is less than *K* in the domain, and then *K* is called Lipschitz constant. This constant specifies the maximum local variation of a continuous function, as shown in Eq (4).


$$\left|f(x_{1})-f(x_{2})|\leq K|x_{1}-x_{2}\right|$$ (4)

If *f* is expressed as a function of parameter *w*, the neural network *f*_*W*_ with weight *W* can be used for simulation. The optimization formula is shown as followed:

$$L=\underset{w\in W}{\max}{\text{E}}_{x∼P_{r}}[f_{w}(x)]-{\text{E}}_{x∼P_{g}}[f_{w}(x)]$$ (5)

However, the weighted clipping can lead to gradient imbalance. As the discriminator is a multi-layer network, if the clipping threshold is small, the gradient will disappear after multiple layers; if the clipping threshold is too large, the phenomenon of gradient explosion will occur. Therefore, gradient penalty (GP) is introduced to strengthen the Lipschitz restriction, that is, an additional Penalty coefficient is set to realize the connection between the gradient and *K*.

In this paper, the "class label" *y* is regarded as the generated "conditional" information, with that GAN model can not only generate the required minority negative samples, but also overcome model collapse. The "class label", noise and real data are input at the same time, thus improving the convergence ability of the model. By this optimization, the model collapse was overcome by adding new constraints and gradient punishment into the discriminator. Then the objective function is translated:

$$\underset{G}{\min}\underset{D}{\max}\underset{\text{Wasserstein}\;\text{loss}}{\underset{︸}{{\text{E}}_{x∼{\text{P}}_{r}}[\text{D}(x|y)]-{\text{E}}_{z∼{\text{P}}_{z}}{\left[\text{D}(G(z|y)|y)\right]}_{w}}}-\lambda \underset{\text{gradient}\;\text{penalty}}{\underset{︸}{{\text{E}}_{\hat{z}∼{\text{P}}_{\hat{z}}}[{\left({\left\|\nabla \text{D}(\hat{z})\right\|}_{w}-1\right)}^{2}]}}$$ (6)

Where, *λ* is the gradient penalty factor; *P*_*penalty*_ is the probability distribution of gradient penalty coefficient;∇ is the gradient. Both of them guarantee that the gradient of discriminator *D* is not to exceed constant *K*. *G*(*z|y*) represents the generated sample of the generator based on the input noise and label *y*, and **D**(*|y*) indicates the probability of determining the label of sample as *y*.

### Stacking ensemble model

**Fig 3:**
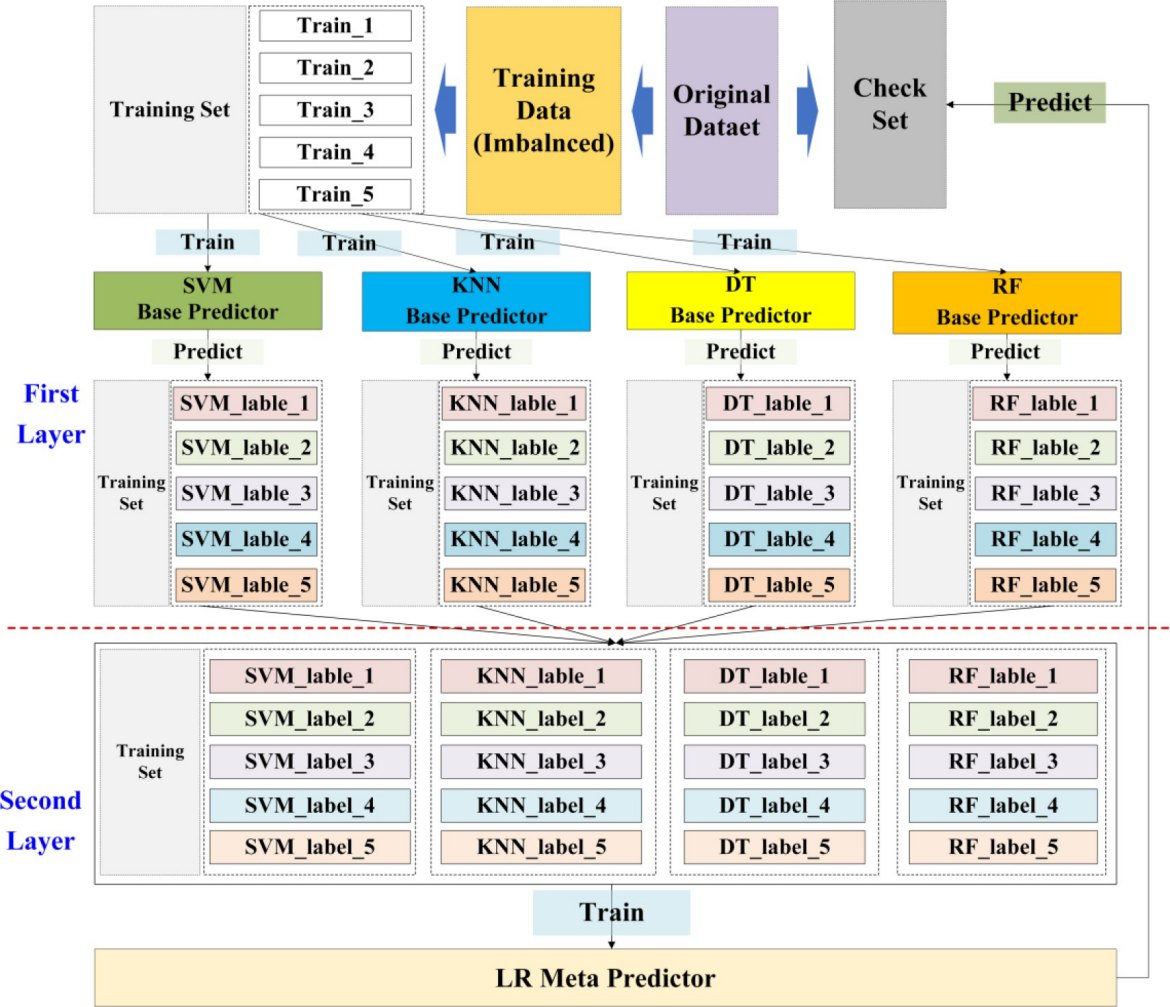
Structure of the two-layer stacking ensemble model.

There are mainly three strategies for ensemble learning: Bagging, Boosting and Stacking. As a label-based ensemble learning strategy, stacking is a multilevel framework based on cross-validation. In this paper, a two-layer stacking ensemble framework consisting of base predictors in the first layer and meta predictor in the second layer with 5-fold cross validation is applied to avoid overfitting (Fig 3).

Four classical stable and simple models, namely SVM, KNN, DT and RF are selected as base predictors. And LR (Logistic Regression) model is chosen as the meta predictor. Training set are divided averagely into five subsets, Train_1, Train_2, Train_3, Train_4 and Train_5, and each subset contains *n* pieces of data. Then, four of them is used to train the train base predictors, and the other one is predicted by the trained base predictor. After 5-fold cross validation operation, all of the training set data are predicted by the base predictor.

**Fig 4:**
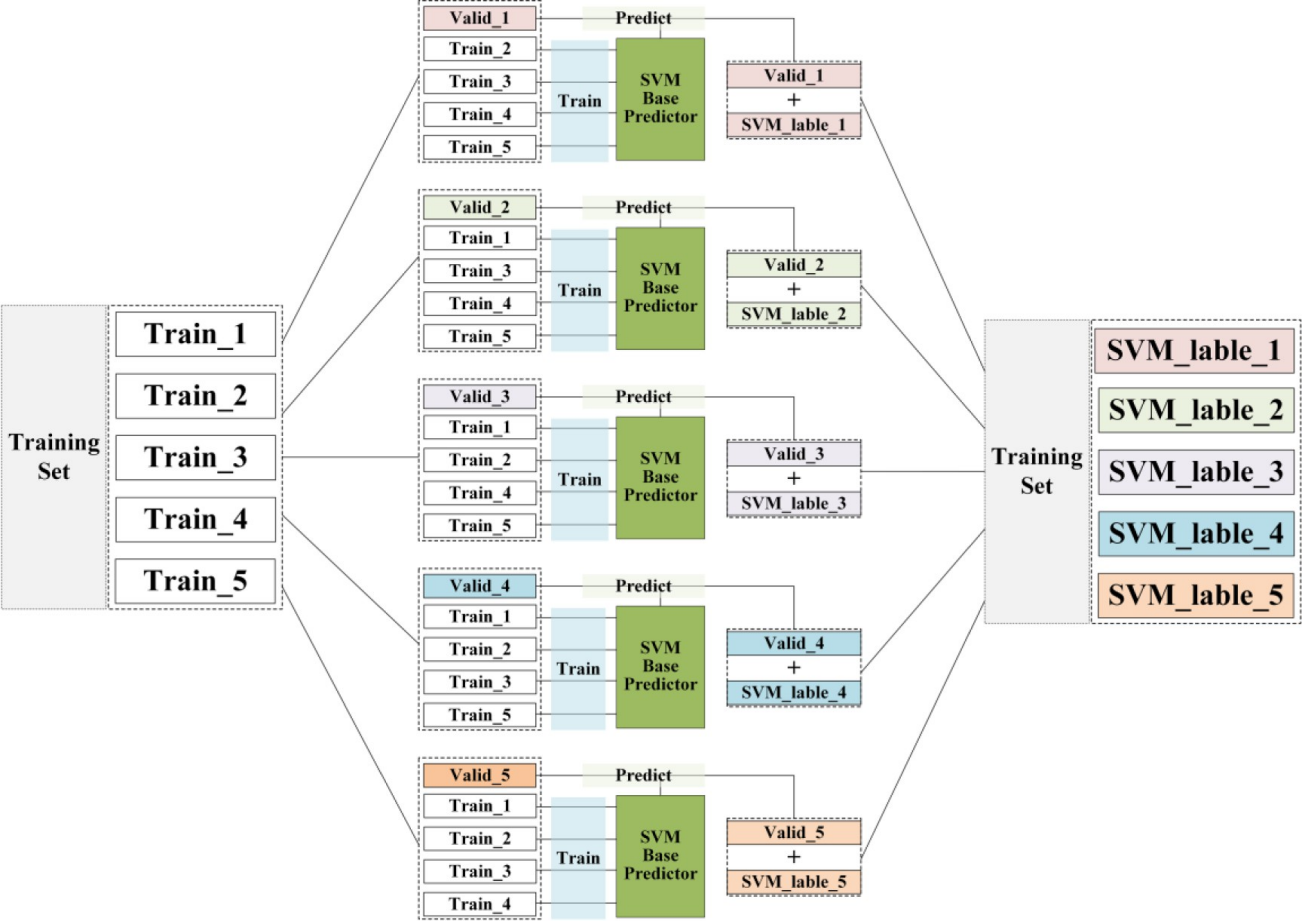
Operation instructions for the first layer of stacking ensemble model.

Taking SVM base predictor as an example, shown in Fig 4. We choose train_1 as validation subset Valid_1 and other subsets Train_2, Train_3, Train_4 and Train_5 as training subsets in the first time. After trained by these 4 training subsets, the SVM base predictor can predict whether the validation set data is anormal or not. If the data is anormal, the output of SVM is 1, otherwise it’s 0. Train_2 is chosen as validation subset Vlid_2 in the second time, while anything else is the same as the first time. Then we can get the SVM predictions for Valid_2. The above procedure is performed five times for 5-fold cross validation. As a result, each data of the training set is predicted by the SVM base predictor, and the result can be treated as a binarized feature: 1 or 0. This feature is a n*1 matrix.

For other three base predictors, KNN, DT and RF, we perform the same operation as for SVM. And each of the base predictors will give us a prediction, that is, other three binarized features: 1 or 0. We further combine these four binary features together for the meta predictor training in the second layer. However, the original data features do not participate in meta predictor training and only the 4 new binary features obtained from base predictors are input into meta predictor model, that is a 5n*4 matrix. As can be seen, the training of the second layer is a simple 4-feature data learning.

Stacking is essentially a kind of representation learning method. In stacking, effective features are learned after passing through multiple learners in the first layer by base predictors. From this perspective, the first layer of stacking is the process of feature extraction. So, the features in the second layer are learned from the data in the first layer. In the process of feature extraction in the first layer by base predictors, we have used a complex nonlinear transformation, so there is no need for a complex classifier in the output layer. In order to overcome the possible overfitting, relatively simple models are generally chosen for stacking ensemble. So, LR model is chosen as the meta predictor, and the output is a probability value instead of a binary function, which is more suitable for subsequent consistency check.

The LR model has probability property and can map sample features to the [0,1] interval. The Sigmoid function used in the mapping process has a very large function gradient and will quickly approach 0/1, which in turn can quickly obtain the probability of different detection results. So LR model is very suitable as the final detection model to score the incremental instances. The prediction function of LR is:

$$y=P(y=1|x;\theta )=h_{\theta }(x)=\frac{1}{1+e^{-\theta^{T}x}}$$ (7)

Where *x* is the data features input into LR model, that is the predictions from the four predictors. It is a 5n*4 matrix. *θ*is the model parameter matrix need to be obtained by training. The meaning of Eq (7) is to calculate the prior probability of the data being anormal under the given conditions of *x* and *θ*. In order to measure whether the best model parameter matrix have been obtained, we defined a cost function based on cross entropy, which is also often used in neural networks. So, the training function of LR model is to obtain the minimum of the cost function:

$$\min J(\theta )\begin{array}{cc} & s.t.\begin{array}{cc} & J(\theta )=-\frac{1}{5n}\left\{\sum_{i=1}^{5n}\left[y^{\left(i\right)}\log h_{\theta }(x^{\left(i\right)})+(1-y^{\left(i\right)})\log(1-h_{\theta }(x^{\left(i\right)}))\right]\right\} \end{array} \end{array}$$ (8)

Where 5*n* is the total number of training data, *x*^(*i*)^ is the input of data *i*, *y*^(*i*)^ is the actual label of the raining data, and *h*_*θ*_(*x*^(*i*)^) is the predicting result based on Eq (7). It can be seen that LR can integrate the advantages of various models so as to realize the secondary learning.

In a nutshell, the data input to the four base predictors in the first layer of Stacking is the rebalanced training data, and the output is the predicting results of each base predictors, that is a binarized feature: 1 or 0. Then, these predicting results from four predictors SVM, KNN, DT and RF are combined together as 4 new features of the training data, to input to the second meta predictor LR (Logistic Regression) in the second layer of Stacking. The output of LR is a probability value of whether the data is anormal.

### Consistency check with statistical learning

Traditional threshold-based detection methods have the problems of parameter sensitivity and experience dependence. Therefore, a new detection method based on consistency check with statistical learning is applied in this paper.

First, the check set *C* = {*z*_1_, *z*_2_, …, *z*_n_} is extracted by random sampling from historical normal data. The size of the validation set is determined according to the volume of historical data and can also be adjusted dynamically according to the incremental rate of the data stream. In this paper, this size is set as a ratio of the historical data. Then, we can score this check set by scoring function, that is, the LR model has been trained in the stacking ensemble model. As a consequence, the check set score sequence can be obtained by arranging the scores of each sample in the check set in ascending order:

$$Seq=\left\{q_{1},q_{2},\ldots ,q_{n-1},q_{n}\right\}\begin{array}{cc} & \left(q_{1}>q_{2}>\ldots >q_{n-1}>q_{n}\right) \end{array}$$ (9)

For a new incremental instance *l*, the scoring function also give its score. The consistency check of the incremental instance with the check set is conducted by comparing their scores. If they are consistent, the incremental instance is treated as a normal sample, otherwise the incremental instance is an anormal one. Traditional threshold-based methods presuppose a real value function for the check set score sequence and new incremental instance. In this paper, the real value function is set as the ranking of the new incremental instance *l* in the check set score sequence, based on the predicting results from LR:

$$S=(C,d)=Ranking(q_{1},q_{2},\ldots ,q_{k-1},l,q_{k}\ldots ,q_{n-1},q_{n})\begin{array}{cc} & \left(k<n\right) \end{array}$$ (10)

Traditional threshold-based methods presuppose a *k* value. If the rank of the incremental instance *l* is higher than *k*, then the incremental instance is more likely to be consistent with the check set, which is normal; otherwise, it is likely to be inconsistent with the check set, which is anormal.

However, the preset ranking position greatly affects the final result, leading to performance fluctuation of anomaly detection, especially under concept drift situation. Therefore, a statistical learning idea is applied, which abandons the traditional threshold-based absolute determination method and use the relative comparison of consistency check results to determine the implied information (normal or anormal). We use *p* value to describe the percentile of the score ranking of the incremental instance in the check set score sequence for relative comparison:

$$p_{i}=\frac{Count\left\{j:s_{i}>s_{j}\right\}}{n}$$ (11)

Where, *n* is the volume of check set; *s*_*i*_ is the score in the check set score sequence, and *s*_*i*_ is the score of the incremental instance. And then we set a confidence level *μ*. When the detection result is higher than this confidence level, it means a higher credibility level for the result.

For example, when *ε* = 0.02, and the *p* value of a new instance is larger than *μ*, then this instance has less than 2% probability to be as inconsistent with the check set. In other words, we have more than 98% certainty that the data is consistent with the check set, namely normal. To obtain the best performance with as little data as possible, the anomaly detection model is not repeatedly trained, and the concept drift is countered by combining with check set obtained during training of confidence levels dynamically updating.

It should be specially noted that the composition of the validation set adopts a put-back sampling method, which means that these normal samples are still retained in the historical data. Besides, as a benchmark for comparison, the check set is dynamically adjusted. New instance with higher score will supplement, so as to keep the check set evolving toward “absolutely normal”.

Usually, the evaluation of anomaly is realized by setting a threshold, that is, when the data exceeds a certain threshold, it is considered as abnormal. However, in this way, the performance of the algorithm will fluctuate for imbalanced data streams with concept drift. On one hand, threshold has strong dependence on data scale, structure and characteristics, and personnel experience also matters. On the other hand, even if a preset threshold in time is appropriate, it may failure with high probability after concept drift occurs.

So, the strategy of consistency detection is adopted. Incremental instance anomaly is detected by statistical learning method, that is the percentile of incremental instance in the validation set is used instead of threshold. Besides, the validation set is dynamic updated according to this percentile result. As a result, it realizes that the anomaly evaluation index changes with the change of data characteristics, and also guarantees the overall anomaly detection accuracy.

## Experimental methodology

### Datasets

Both real-world data streams and synthetic data streams are used in these experiments. The synthetic data streams are used to compare the performances of the proposed method under specific conditions. In terms of data generation, the imbalance ratio, location, number, and amplitude of drift were set to obtain experimental data based on MOA (Massive Online Analysis) platform. In addition, some of real datasets are selected as historical data, and the rest data are selected as incremental instances according to data entries for the experiments.

Three artificial data sets, SEA, RBF and Waveform, are generated by MOA platform. MOA was developed by the Machine Learning Group at the University of Waikato based on the WEKA (Waikato Environment for Knowledge Analysis) extension project. A variety of artificial and real data flow generators are provided, which can be easily formulated according to requirements parameters generate a data stream. For example, the ArffFileStream generator can simulate the static real data to generate data flow. At the same time, under MOA, the data flow generator can also be used to simulate the generation of steams containing different concept drifting types. In addition, data streams with different labels can also be generated for anomaly detection studies. The proportion of anormal data can be set manually, which is related to the imbalance ratio. SEA is an abrupt drift data set, including three numerical features, generated by SEA Drift Generator; RBF is an abrupt and gradual drift hybrid data set, generated by Random RBF Drift Generator; Waveform is an abrupt drift data set, generated by Waveform Drift Generator.

Other two real-word datasets are also used: 1) HDFS, the original Hadoop distributed file system log data collected by The University of Berkeley, 1.58GB in total, containing 11,197,705 original logs. We random sampled 10% of them including 16,838 anormal samples, manually marked by domain experts, involving 580 features, and the proportion of anormal data is 3.6%. 2) Spam, a gradual drift data set containing 9324 email instances. Among them, spam mails are marked as anormal. The above data sets were used for experiments, as shown in Table 1.

**Table 1:** Experimental data sets.

Dataset	Number of instances	Number of class values	Number of drift point	Concept drift type	Imbalance ratio
SEA	1,000,000	4	3	Abrupt	5/10/20/100
RBF	1,000,000	2	5	Hybrid	5/10/20/100
Waveform	1,000,000	3	3	Gradual	5/10/20/100
HDFS	1,119,770	2	-	-	34
Spam	9234	2	-	-	55

### Baseline methods

In order to evaluate the effectiveness and superiority of the proposed model. The proposed was compared with three types of baseline methods, typical concept drift detection methods such as accuracy weighted ensemble (AWE), dynamic weighted majority (DWM), leveraging bagging (Lev Bagging) and online dynamic updated ensemble (OAUE). AWE is an ensemble algorithm based on the accuracy weighted. DWM is a dynamic weighted majority voting algorithm. Lev Bagging is a Bagging online ensemble algorithm. OAUE is based on the cycle weighting for online processing.

Typical imbalance related techniques considering imbalanced data and concept drift issues comprehensively are also used to make comparisons, such deterministic sampling classifier with weighted bagging (DSCB) [39] and dynamic updated ensemble (DUE) [40].

The experiments were executed on a machine equipped with an eight-core Intel i9 CPU, a 3.4 GHz processor and 16 GB of RAM. All experiments were repeated ten times with the random seeds set from 1 to 10. The results shown are the average values of ten trials.

### Evaluation metrics

To verify the performance of the proposed model, the experimental verification and analysis are carried out from four aspects: detection performance, parameters sensitivity, algorithm cost and anti-noise ability.

For detection performance, detection accuracy rate is chosen for imbalanced data measure. For parameters sensitivity, two parameters of the ratio of check set and the confidence level are taken into consideration. Because, with they are directly determines the result of consistency check and anomaly detection. Furthermore, in order to compare the cost of the proposed method, the time and memory consumed by different algorithms are compared. Noise mainly affects detection accuracy and has impact on space-time consumption. Since the noise situation in the real dataset cannot be determined, the anti-noise study of these algorithms is mainly carried out based on the synthetic datasets.

## Results and analysis

### Detection performance

**Fig 5:**
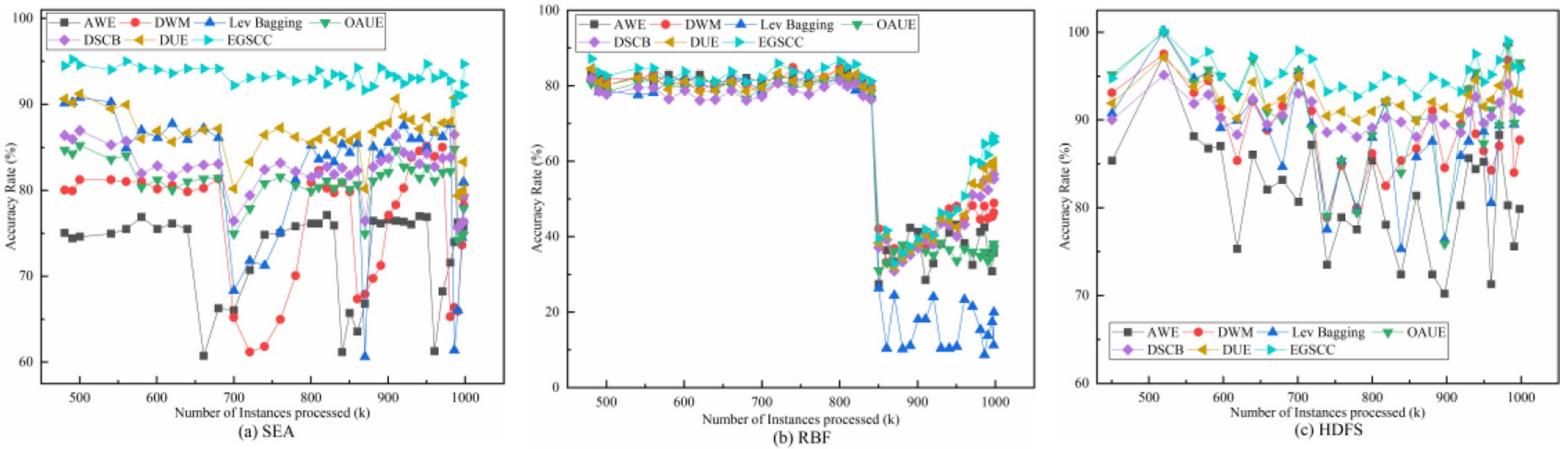
Accuracy rate with the number of instances processed.

Fig 5 shows the quantitative relationship between the accuracy rate of different algorithms and the number of processed instances. The position and fluctuation of curves indicate the adaptability of different algorithms to concept drift.

Fig 5(A) shows that abrupt concept drifts occurred three times on SEA dataset (around 550K, 700K and 875K, respectively), and the accuracy rate of each algorithm changed significantly with different delays after concept drifts occurred. From the perspective of curve fluctuation, with the increase of the number of instances processed, various algorithm models have different degrees of concept drift, which is reflected in the significant decrease of the accuracy rate. Compared with the other six methods, the accuracy rate of the proposed EGSCC method is always beyond 95%, and its fluctuation is very small when concept drift occurs, so it can quickly recover to the original accuracy. Other algorithms such as AWE are less effective for abrupt drift, so the overall adaptability to concept drift is limited, and the accuracy decreases more obviously and fluctuates more.

The reason for the poor performance is not only concept drift but also data imbalance, so algorithms only dealing with concept drift perform as well as any other algorithms dealing with the two issues together, such as DSCB, DUE and the proposed EGSCC.

Fig 5(B) shows the relationship between accuracy rate and number of instances processed on RBF dataset. The experimental results show that the accuracy of each method is excellent and stable before the number of processing cases is 850K, and all of them exceed 80%. Significant concept drift occurred at the number of processed instances 850K, and the accuracy rate of each model significantly decreased to 25%-40% and kept low fluctuations. While the accuracy rate of the proposed EGSCC method rebounded to 70% after a period of adjustment, which is significantly higher than other methods, followed by DSCB and DUE. The recovery is not obvious for method only dealing with concept drift. The case of imbalanced data is even more serious after concept drift, which may also lead to feature overlap, so the accuracy rate dramatic declines for algorithms such as AWE, DWM.

The results of the accuracy rate variation of each algorithm in the HDFS dataset are shown in Fig 5(C). The curves fluctuate several times indicating that concept drift occurs. However, compared with other algorithms, the accuracy rate of the proposed EGSCC method always stays above 95% with the least fluctuation, which means a better combined performance of accuracy and stability.

In contrast to DSCB and DUE, dealing with the imbalance and concept drift together, the stacking ensemble model adopted by EGSCC can quickly capture the abrupt concept drift and the probability calculation of the consistency check can also achieve the effect of coping with the gradual concept drift. Furthermore, it can effectively adapt to multiple types of hybrid concept drift. Table 2 shows the statistical results of the average accuracy rate of each algorithm on different datasets.

**Table 2:** Statistical results of the accuracy rate.

Data Set	AWE	DWM	Lev Bagging	OAUE	DSCB	DUE	EGSCC
SEA	75.59 (7)	85.10 (6)	89.93 (4)	86.85 (5)	90.75 (3)	91.43 (2)	**95.12 (1)**
RBF	55.19 (7)	58.27 (6)	65.89 (5)	63.47 (4)	70.83 (2)	67.22 (3)	**71.78 (1)**
Waveform	85.32 (5)	82.63(6)	90.64 (2)	80.17 (7)	87.46 (4)	**90.88 (1)**	88.25 (3)
HDFS	85.74 (7)	90.52 (4)	90.37 (5)	91.54 (2)	91.17 (3)	89.89 (6)	**95.26 (1)**
Spam	78.22 (6)	76.41 (7)	85.20 (5)	**89.74 (1)**	86.02 (3)	85.52 (4)	86.45 (2)
**Average**	76.01 (7)	78.59 (6)	84.41 (4)	82.35 (5)	85.25 (3)	84.99 (2)	**87.37 (1)**

Table 2 shows that EGSCC has significant advantages over other algorithms on SEA and RBF artificial datasets (ranked 1st) and achieves acceptable result on Waveform dataset (ranked 3rd) and Spam (ranked 2nd). It indicates that the proposed EGSCC method is more adaptable to abrupt drift. In addition, EGSCC has the best overall performance on the five experimental datasets with an average accuracy rate of 87.37%, ranking first; the DUE method comes secondly with an average accuracy rate of 84.99%; DSCB ranks thirdly with an accuracy rate of 85.25%; the Lev Bagging method has the fourth overall performance with an accuracy rate of 84.41%; and the AWE method has the worst overall performance with an accuracy rate of 76.01%.

Further analyzing the performance of the EGSCC method on two real-word datasets, it ranks 1st on the HDFS dataset and 2nd on the Spam dataset, indicating that the proposed method is also very effective on real-word datasets. The accuracy rate of EGSCC is slightly lower than OAUE on the Spam dataset, however it is an online detection mechanism with higher learning efficiency and less memory consumption. Therefore, the comprehensive performance of EGSCC method is optimal, and the smaller data volume of Spam data set has more demanding requirements on the learning ability of the algorithm. In addition, ensemble learning method including DSCB, DUE and the proposed EGSCC perform better than non-ensemble methods, because of the simultaneous coping with imbalance and concept drift

**Fig 6:**
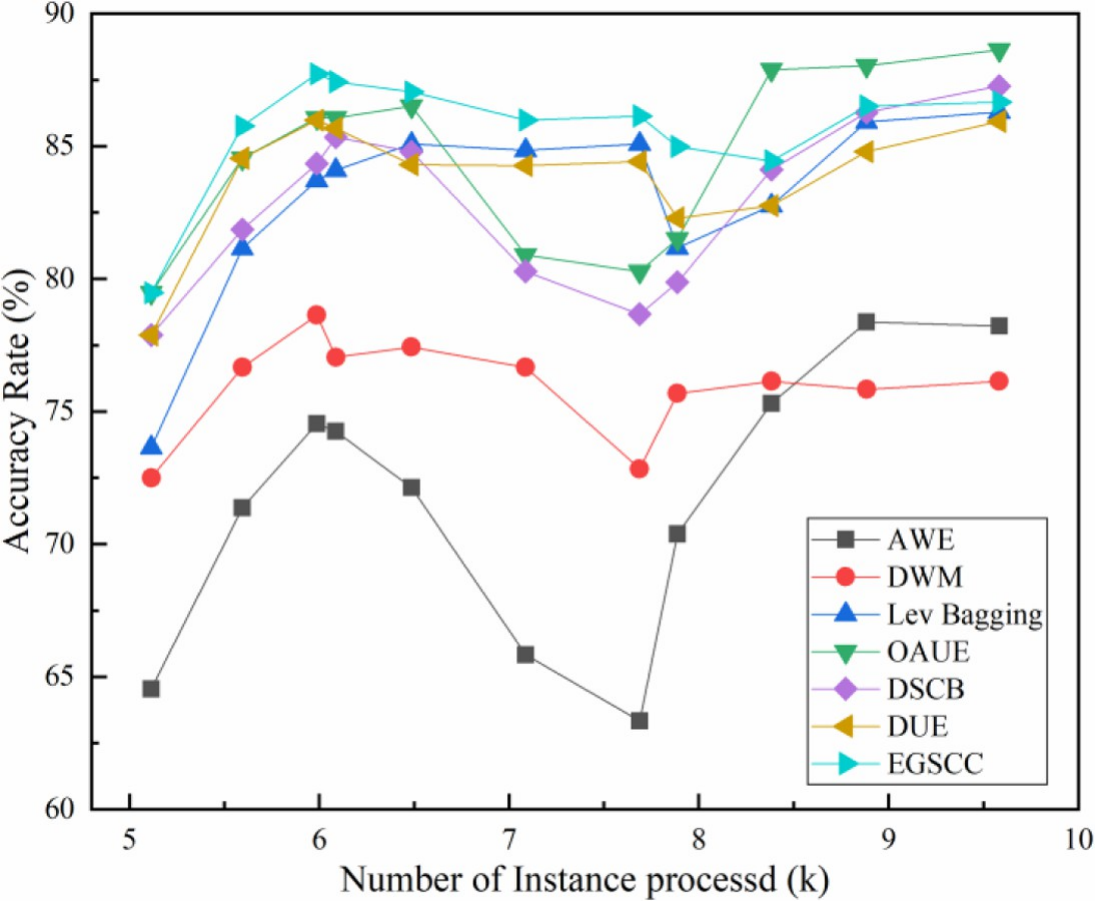
Accuracy rate comparison on Spam dataset.

The results on the Spam data set are shown in Fig 6. It shows that on the Spam dataset, the highest accuracy rate of the EGSCC algorithm is above 85%, when incremental instances are less than 8k. And it remains a good performance when the increment instance is more than 8k. However, the accuracy rate of OAUE rises from 80% to nearly 90%, and the EGSCC algorithm ranks the second. In terms of stability analysis, the EGSCC algorithm is undoubtedly the best, while the OAUE algorithm performs better when the number of processed instances is smaller (<6.5k) or when the number of processed instances is higher, while the performance fluctuates when the number of processed instances is in the median. Other two ensemble learning method DSCB and DUE also shows advantages in terms of stability. These results show that the EGSCC proposed in this paper performs better and balanced throughout the training phase. Meanwhile, as a real data set, there is a hybrid of multiple types of drift in Spam, and the accuracy and stability advantages of EGSCC algorithm also reflect its good adaptability to hybrid drift.

### Parameters sensitivity

The EGSCC algorithm proposed in this paper focuses on the classification by statistical learning rather than threshold. So, the anomaly detection result is affected by two important parameters: the ratio of check set *r* and confidence level *μ*. The experiment results of EGSCC under different ratios of check set *r* are shown in Table 3.

**Table 3:** Effect of ratio of check set on detection accuracy rate.

Ratio of check set *r* (%)	5	10	15	20
SEA	90.1	**95.1**	88.7	93.1
RBF	87.8	90.4	**90.7**	87.7
Waveform	89.6	**93.2**	89.6	92.2
HDFS	88.4	**91.2**	87.3	90.2
Spam	88.5	**90.3**	87.4	88.9

As shown in Table 3, the EGSCC model achieves the highest accuracy in four of the five experimental datasets when the ratio of check set *r* is around 10%, and the accuracy in the SEA dataset is slightly less than that when the ratio of check set *r* is 15%. However, the difference between these two results is not significant, so *r* = 10% can be used as the best parameter of the ratio of check set under the experimental conditions. The EGSCC algorithm significantly improves the overall detection performance by taking into account both accuracy and variability.

**Fig 7:**
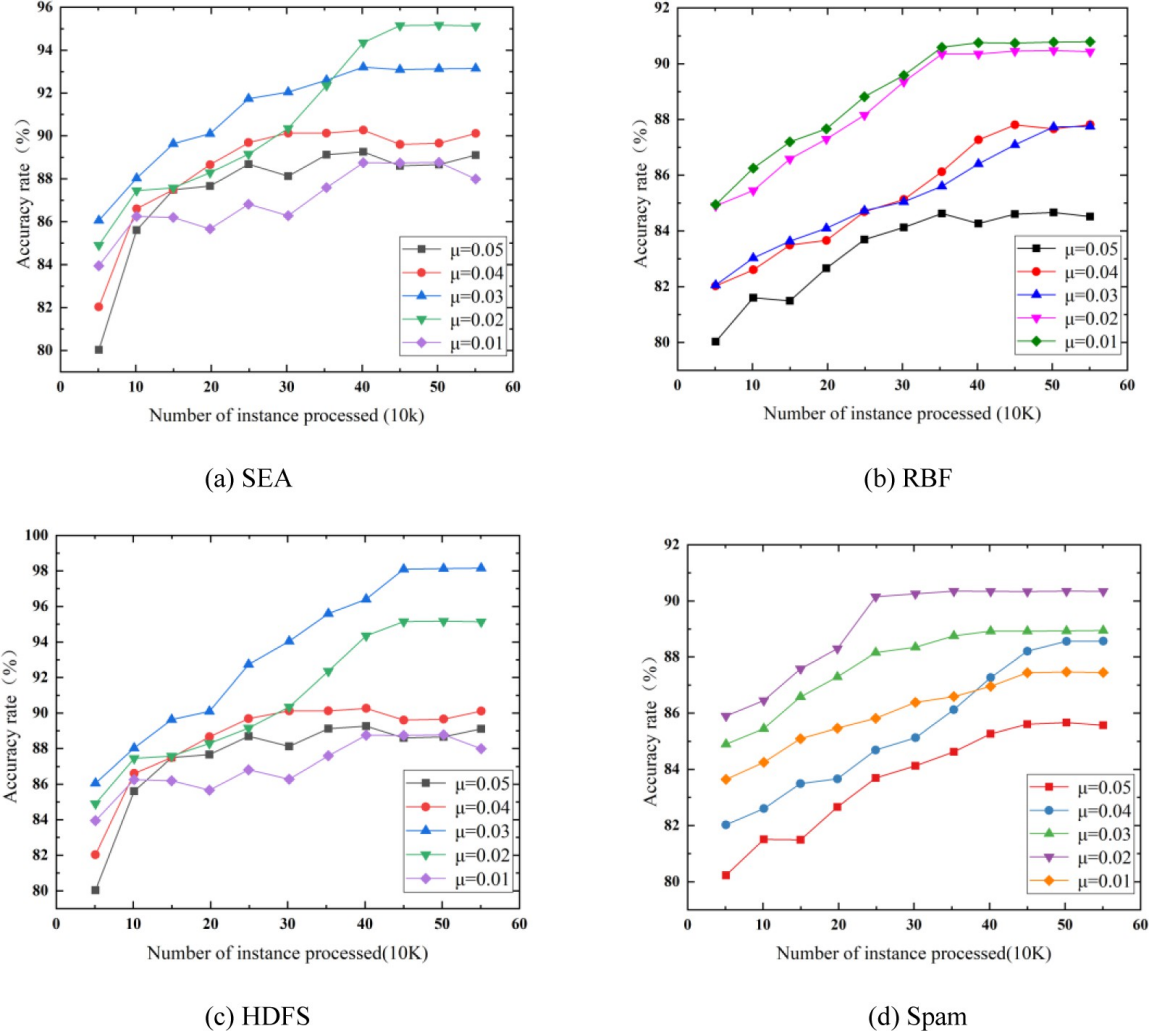
Effect of confidence level on detection accuracy rate. (a) SEA. (b) RBF. (c) HDFS. (d) Spam.

Fig 7 shows the accuracy rate of EGSCC model with different confidence level *ε*. SEA dataset is taken as an example to compare the accuracy rate of EGSCC with incremental instances under different confidence level *μ*. As can be seen from, with the increase of the number of incremental instances, the detection accuracy rate of EGSCC increases gradually and reaches a stable level without large fluctuations. It shows a good learning ability for incremental instances without obvious concept drift. Meanwhile, comparing the detection accuracy rate under different confidence levels, we can see that *μ* = 0.02 has the best overall detection accuracy rate on the SEA dataset, which is also proved by the experimental results on several other datasets.

### Algorithm cost

Furthermore, in order to test the cost of EGSCC algorithm running on different data sets, the time and memory consumed by different algorithms are compared. The comparison results of computational efficiency are shown in Table 4.

**Table 4:** Comparison of running time consumption of each algorithm (seconds).

Data set	AWE	DWM	Lev Bagging	OAUE	DSCB	DUE	EGSCC
SEA	**11.75 (1)**	32.23 (6)	42.29 (7)	13.60 (4)	12.79 (3)	13.68 (5)	12.12 (2)
RBF	14.94 (3)	17.14 (4)	28.89 (5)	**8.72 (1)**	10.58 (2)	14.13 (4)	13.94 (3)
Waveform	6.19 (3)	**4.31 (1)**	12.56 (6)	18.47 (7)	6.97 (5)	6.28 (4)	5.44 (2)
HDFS	18.94(6)	14.24 (2)	24.41 (7)	**10.89 (1)**	17.54 (4)	18.03 (5)	16.80 (3)
Spam	**2.31 (1)**	2.61 (4)	2.99 (7)	2.79 (5)	2.45 (3)	2.77 (6)	2.42 (2)
**Average Ranking**	2.8 (2)	3.4 (3)	6.4 (7)	3.6 (5)	3.4 (3)	4.8 (6)	**2.4 (1)**

The experimental results show that the proposed EGSCC algorithm has the highest average ranking of time consumption and the best comprehensive computational efficiency on five data sets. And the AWE algorithm has slightly inferior comprehensive computational efficiency on five data sets than EGSCC, however, the difference between them is not obvious. And its computational performance exceeds that of EGSCC algorithm on two other data sets, SEA and Spam. The combined computational efficiency of the DWM algorithm and the OAUE algorithm is comparable, and the former having an advantage on Waveform datasets, while the latter is more applicable to HDFS datasets. The Lev Bagging algorithm has the largest computational consumption. There is a big gap between DSCB and DUE. The average potential ranking of DSCB is 3.4, which is the third among the seven algorithms, while DUE has an average ranking of 4.8, which is only better than Lev Bagging. The EGSCC algorithm uses the detection mechanism of consistency check to avoid unnecessary model updates; meanwhile, the GAN-based resampling strategy and of stacking ensemble achieves efficient and more reasonable model screening and avoids unnecessary computations.

Table 5 shows the memory consumption of each algorithm. EGSCC consumes the least memory, followed by AWE, while Lev Bagging is the costliest. The experimental results show that the proposed DWM algorithm has the minimal average ranking of memory consumption on five data sets. And the proposed EGSCC algorithm has slightly inferior comprehensive memory consumption on five data sets than DWM. And it exceeds that of DWM algorithm on HDFS dataset. The OAUE algorithm has the largest memory consumption. There is some difference between DSCB and DUE. The average potential ranking of DSCB is 3.8, which is the fourth among the seven algorithms, while DUE has an average ranking of 4.6, which is next to the DSCB. Due to the fact that it does not establish a detection model for each new instance and does not reconstruct the model periodically, EGSCC reduces the memory consumption of frequent modeling.

**Table 5:** Comparison of memory consumption of each algorithm (MB).

Data Set	AWE	DWM	Lev Bagging	OAUE	DSCB	DUE	EGSCC
SEA	2.68 (3)	**1.11 (1)**	37.30 (7)	6.83 (6)	4.95 (4)	5.77 (5)	2.14 (2)
RBF	10.76 (7)	**1.04 (1)**	3.61 (5)	7.62 (6)	3.07 (4)	2.13 (2)	2.23 (3)
Waveform	**5.05 (1)**	6.18 (2)	80.29 (7)	30.71 (6)	7.96 (4)	9.41 (5)	6.37 (3)
HDFS	6.23 (3)	9.31 (6)	7.27 (7)	13.46 (6)	6.14 (2)	8.55 (5)	**4.26 (1)**
Spam	0.84 (2)	**0.60 (1)**	1.46 (3)	1.94 (7)	1.66 (5)	1.89 (6)	1.63 (4)
**Average ranking**	3.2 (3)	**2.2 (1)**	5.8 (6)	6 (7)	3.8 (4)	4.6 (5)	2.6 (2)

**Fig 8:**
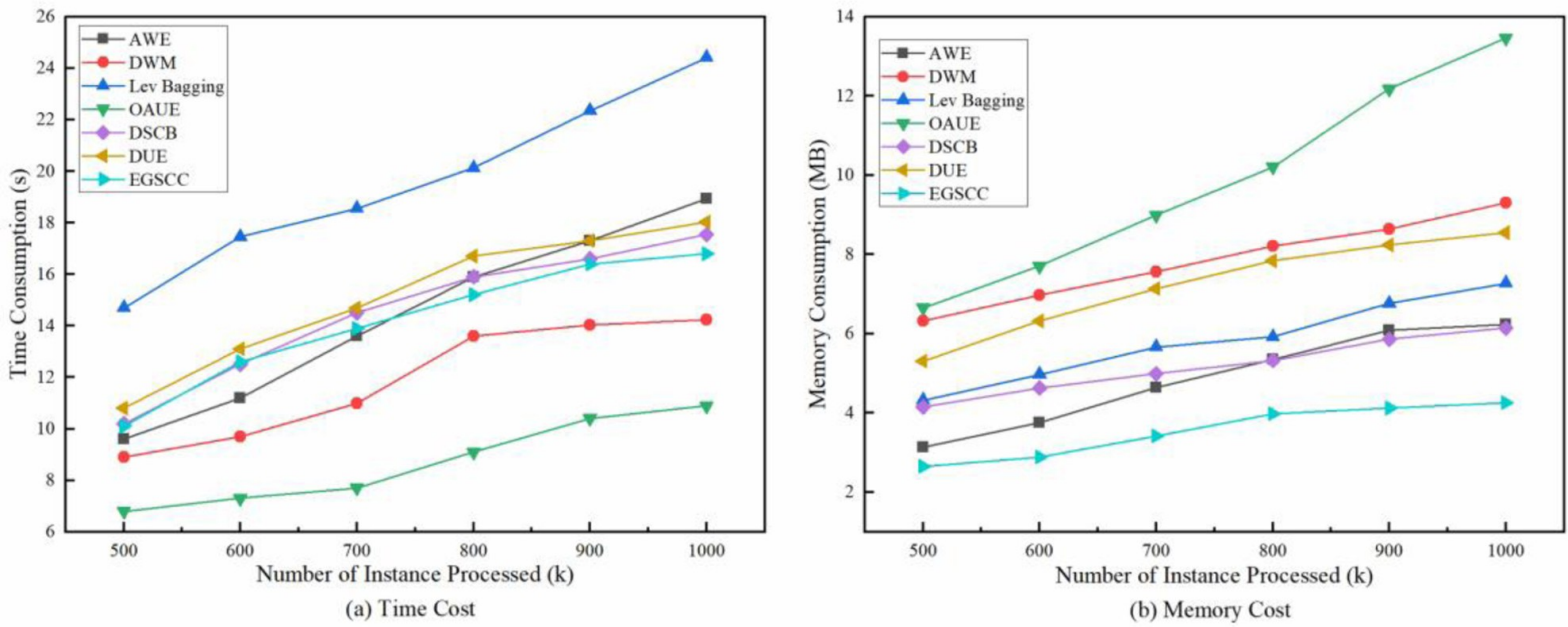
Asymptotic complexity of algorithm in terms of time and memory cost on HDFS dataset.

An asymptotic complexity of the proposed algorithm compared with the baseline methods in terms of execution time and memory cost on HDFS dataset is provided. And the results are shown in Fig 8, showing that ESSGHC has a better comprehensive advantage of time and memory consumption.

As shown in Fig 8(A), Lev Bagging is obviously the most time-consuming and OAUE is the obviously the most time saver. Because Lev Bagging uses the strategy of refactoring the model, so it requires constant repetition of training the detector, which is time-consuming. And for OAUE, it employs a strategy of dynamic selection, that is, once the predictors are trained, they do not need to repeat the training, and only need to perform simply selecting. Other methods including the proposed EGSCC use ensemble learning strategy, whose time cost is between model refactoring and model optimization. In addition, EGSCC has no obvious advantage when the amount of data is small, and DUE seems more advantageous. When the amount of data continues to increase, its advantages gradually show, due to the representation learning ability of Stacking from heterogeneous models. In Fig 8(B), it can be seen that OAUE is obviously the most memory-consuming and the proposed EGSCC is the obviously the most time saver. Dynamic selection strategy of OAUE makes it necessary to stores the parameters of all models, which is the most memory-consuming. As the amount of data increases, the memory consumption of each algorithm does not increase significantly. And the proposed EGSCC always keeps memory consumption to a minimum.

### Anti-noise ability

**Fig 9:**
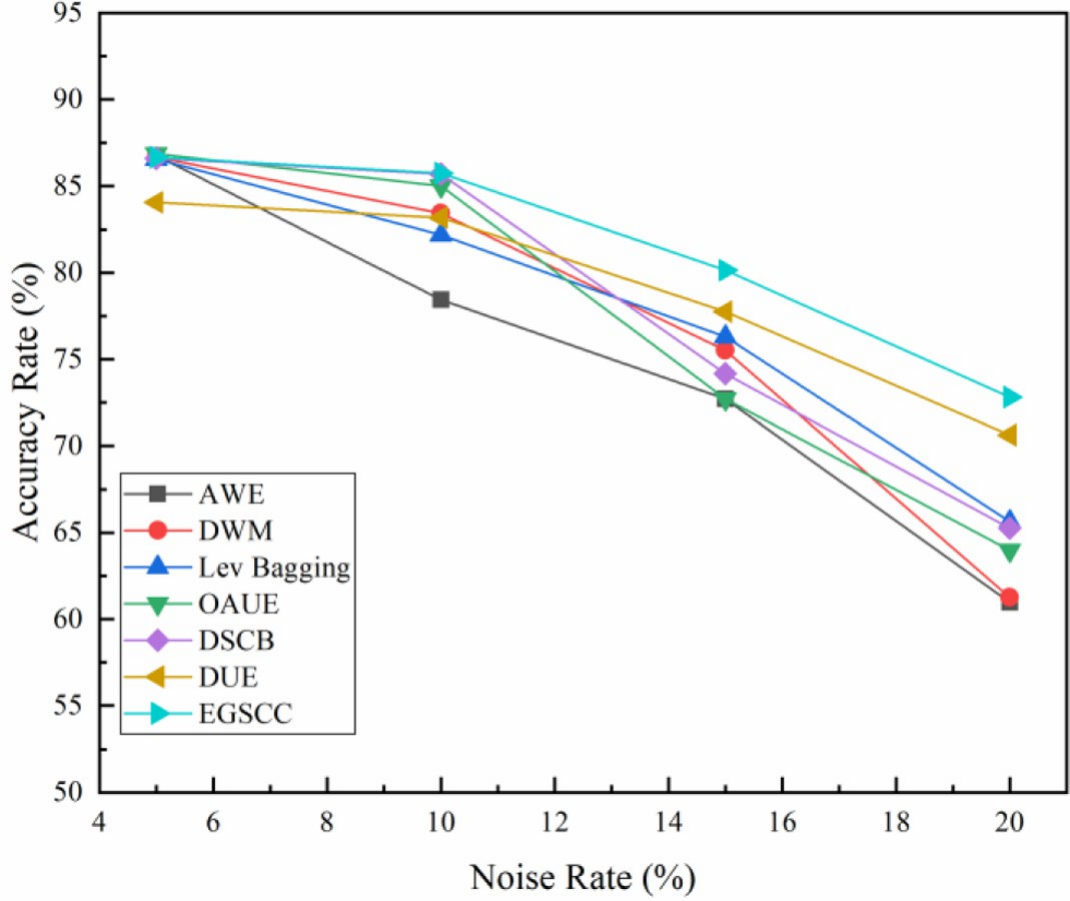
Accuracy rate under different noise rates on SEA dataset.

Noise mainly affects detection accuracy and has less impact on space-time consumption. Since the noise situation in the real dataset cannot be determined, the anti-noise study of the algorithms is mainly carried out based on the synthetic dataset. Fig 9 shows the accuracy of each algorithm versus noise rate on the SEA dataset.

Experimental result show that the higher the noise rate, the lower the accuracy rate. Especially when the noise rate exceeds 10%, the accuracy of the all algorithms decreases significantly and the detection performance degrades seriously. When comparing the five different algorithms horizontally, the accuracy of EGSCC algorithm decreases with the increase of noise rate, however it is still higher than the other four algorithms, especially when the noise rate exceeds 10%.

**Fig 10:**
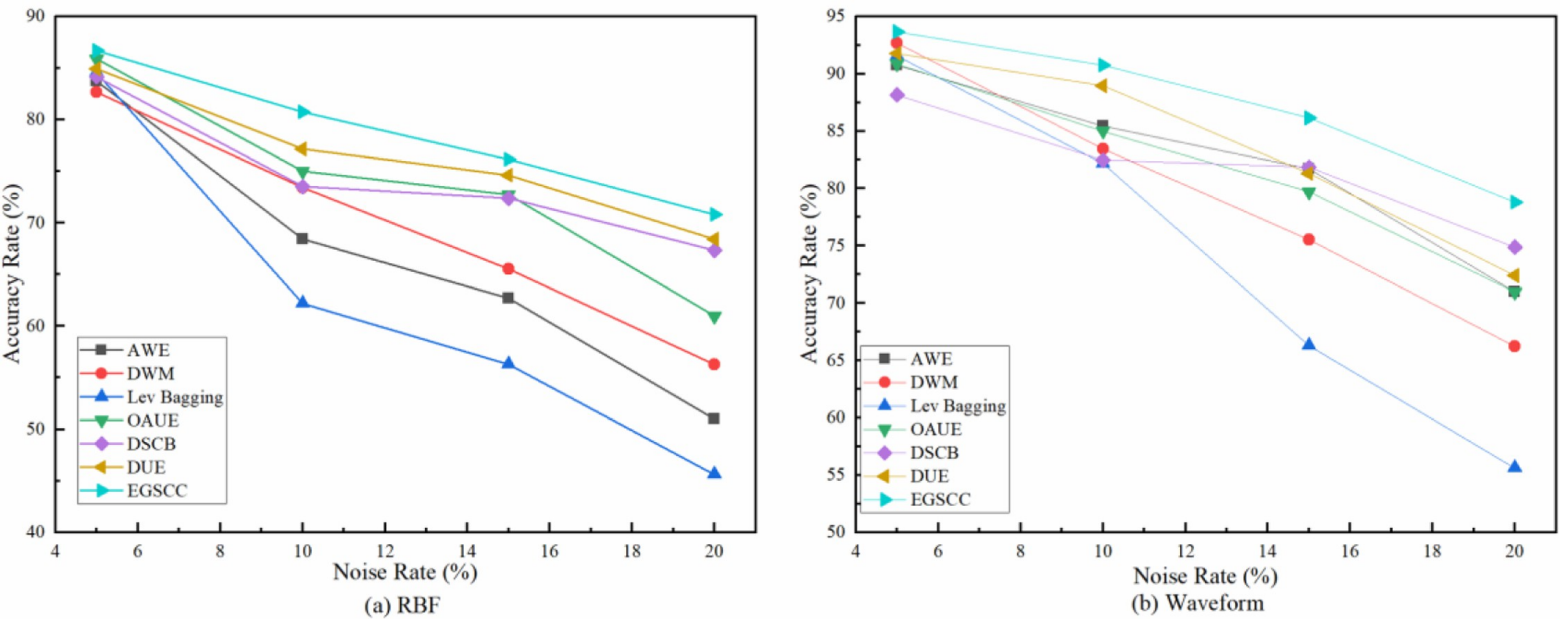
Accuracy rate under different noise rates on RBF and Waveform datasets.

Accuracy advantage of EGSCC becomes more obvious in higher noise rate, and the accuracy rate always remains above 75%, which reflecting its advantages in noise resistance. Further analyzing the reason, GAN-based oversampling, stacking ensemble learning and consistency check with statistical learning ensure the robustness of EGSCC, so that it has better anti-noise performance. This rule has also been verified in RBF and Waveform datasets, as shown in Fig 10.

## Conclusion

In this paper, we discuss the issues of concept drift and class imbalance. While each of these two problems has been well studied, the combined issue is mostly explored even though it has attracted enough attention because of its wide real-world applications. EGSCC is proposed to solve the problem of anomaly detection for imbalanced data streams with concept drift. It combines the advantages of oversampling, ensemble learning and statistical learning. An optimized GAN model with double encoders is introduced for oversampling first, dealing with imbalance problem. Then, the stacking ensemble framework with 5-fold cross validation is undertaken to train the score function for anomaly detection. Furthermore, we undertake consistency check of incremental instances with the check set. And the detection result is obtained by the percentile of incremental instance in the validation set, instead of threshold. Finally, experiments about detection performance, parameters sensitivity, algorithm cost and anti-noise ability have been carried out. And the results verify the better performance of the proposed EGSCC method.

The EGSCC method introduced in this paper mainly focuses on binary classification in anomaly detection, however, it could be easily extended to multi-class issues. Besides, class overlap often occurs with class imbalance, which is not involved in this paper. So, directions for future work include applying the proposed framework to multi-class data streams with skewed class distributions and complex data distributions.

## Supporting information

S1 Fig Accuracy rate with the number of instances processed. (OPJU)

S2 Fig Accuracy rate comparison on Spam dataset. (OPJU)

S3 Fig Effect of confidence level on detection accuracy rate (SEA). (OPJU)

S4 Fig Effect of confidence level on detection accuracy rate (RBF). (OPJU)

S5 Fig Effect of confidence level on detection accuracy rate (HDFS). (OPJU)

S6 Fig Effect of confidence level on detection accuracy rate (Spam). (OPJU)

S7 Fig Asymptotic complexity of algorithm in terms of time and memory cost on HDFS dataset. (OPJU)

S8 Fig Accuracy rate under different noise rates on SEA dataset. (OPJU)

S9 Fig Accuracy rate under different noise rates on RBF and Waveform datasets. (OPJU)

S1 Table Statistical results of the accuracy rate. (XLSX)

S2 Table Effect of ratio of check set on detection accuracy rate. (XLSX)

S3 Table Comparison of running time consumption of each algorithm (seconds). (XLSX)

S4 Table Comparison of memory consumption of each algorithm (MB). (XLSX)

S1 File Data availability statement. (DOCX)
