# Supplementary material for: An ensemble learning method with GAN-based sampling and consistency check for anomaly detection of imbalanced data streams with concept drift
Source: PLoS One. 2024 Jan 26;19(1):e0292140. doi: 10.1371/journal.pone.0292140 (PMC10817223; doi:10.1371/journal.pone.0292140)
Supplement: S1 File — (DOCX) [file pone.0292140.s014.docx]

Data Availability Statement

Aug. 22^th^, 2023

Dear Editors,

We would like to submit the enclosed manuscript entitled “An ensemble learning method with GAN-based sampling and consistency check for anomaly detection of imbalanced data streams with concept drift”, which we wish to be considered for publication in “PLOS ONE”. The authors are from China.

The minimal data set underlying the results described in our manuscript can be found from the “Data” file in this Supporting Information file.

As for the code sharing, we are so sorry to share our code without restrictions. Because this paper is supported by the Fundamental Research Funds for the Central Universities of Civil Aviation University of China (grant no. 3122023033). We have promised that all codes would be treated as research achievement delivered to the funder, and could be disclosed to third parties in public without permission. However, if editors and reviewers want to access the code, they can contact the author individually for the code. We regret it again and hope that it will not seriously affect the publication of this manuscript.

Sincerely

He Sui

College of Aeronautical Engineering, Civil Aviation University of China,

Jinbei Road No.2898, Dongli District, Tianjin 300300, China.

Phone: +86-22-2409-2294, Fax: +86-22-2409-2294, Email: hsui@cauc.edu.cn
